# Significance of fecal hemoglobin concentration for predicting risk of colorectal cancer after colonoscopy

**DOI:** 10.1002/jgh3.12346

**Published:** 2020-04-16

**Authors:** Takuji Kawamura, Takato Inoue, Ryo Shinomiya, Hiroaki Sakai, Kana Amamiya, Naokuni Sakiyama, Atsushi Shirakawa, Yusuke Okada, Kasumi Sanada, Kojiro Nakase, Koichiro Mandai, Azumi Suzuki, Mai Kamaguchi, Atsushi Morita, Kenichi Nishioji, Kiyohito Tanaka, Koji Uno, Isao Yokota, Masao Kobayashi, Kenjiro Yasuda

**Affiliations:** ^1^ Department of Gastroenterology Kyoto Second Red Cross Hospital Kyoto Japan; ^2^ Department of Health Care Kyoto Second Red Cross Hospital Kyoto Japan; ^3^ Department of Biostatistics, Graduate School of Medicine Hokkaido University Sapporo Japan

**Keywords:** cancer screening, colonoscopy, colorectal cancer, occult blood

## Abstract

**Background and Aim:**

As the significance of the quantitative fecal immunochemical test (FIT) in patients who previously underwent a colonoscopy is unknown, this study aimed at investigating the association between fecal hemoglobin concentration and the risk of colorectal cancer (CRC).

**Methods and Results:**

We retrospectively analyzed FIT‐positive patients who underwent a colonoscopy through our opportunistic annual screening program from April 2010 to March 2017 at the Kyoto Second Red Cross Hospital. We stratified them into no colonoscopy and past colonoscopy (>5 years or ≤5 years) groups based on whether they had a history of undergoing a colonoscopy and analyzed the correlation between fecal hemoglobin concentration and advanced neoplasia or invasive cancer detection in each group. We analyzed 1248 patients with positive FIT results. There were 748 (59.9%), 198 (15.9%), and 302 (24.2%) patients in the no colonoscopy, past colonoscopy (>5 years), and past colonoscopy (≤5 years) groups, respectively. In the no colonoscopy group, the advanced neoplasia detection rate significantly increased with the fecal hemoglobin concentration (*P* < 0.001). However, no significant trend was observed in the past colonoscopy (both >5 years and ≤5 years) group (*P* = 0.982). No invasive cancer was detected in the past colonoscopy (≤5 years) group.

**Conclusion:**

The risk of CRC might be low even if fecal hemoglobin concentration was high, especially in those who underwent colonoscopy within 5 years.

## Graphical abstract

We retrospectively analyzed fecal immunochemistrytest positive patients who underwent a colonoscopy. We stratified them into the no colonoscopy and past colonoscopy (>5 years or ≤5 years) groups based on whether they had a history of undergoing a colonoscopy and analyzed the correlation between fecal hemoglobin concentration and advanced neoplasia detection in each group. In the no colonoscopy group, the advanced neoplasia detection rate significantly increased with the fecal hemoglobin concentration. However, no significant trend was observed in the past colonoscopy (both ≥5 years and ≤5 years) group. Especially in those who underwent colonoscopy within 5 years, risk of colorectal cancer might be low even if fecal hemoglobin concentration was high.

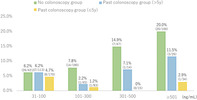

## Introduction

Colorectal cancer (CRC) screening using the fecal occult blood test effectively reduces mortality from the disease.^1^, ^2^, ^3^, ^4^, ^5^, ^6^ In recent years, these tests are often performed using the immunochemical method (fecal immunochemical test [FIT]),^6^, ^7^ and a total colonoscopy is recommended when a FIT result is positive.^8^ In the Japanese population‐based CRC screening program, an annual 2‐day FIT method is used according to the guidelines recommended by the Ministry of Health, Labour and Welfare,^9^, ^10^ and repeating annual FIT is recommended even after colonoscopic intervention.

However, the value of repeating FIT following a colonoscopy remains controversial. In Japan, repeating annual FIT is often performed according to the recommendations of the Ministry of Health, Labour and Welfare, even 1 year after negative colonoscopy. Nevertheless, we have reported the risk of CRC or advanced neoplasia (defined as any tubular adenoma measuring ≥10 mm or with features of villous histology, high‐grade dysplasia, or invasive cancer) to be low in FIT‐positive, average‐risk patients subjected to colonoscopy within 5 years in our Japanese hospital.^11^ According to the European Society of Gastrointestinal Endoscopy guidelines, if there is no high‐risk lesion in colonoscopy, rescreening with FIT or colonoscopy is recommended after 10 years.^12^ Furthermore, it is not recommended to repeat FIT within 10 years; however, if the FIT is found to be positive within 10 years unexpectedly, it is up to the clinician to decide whether to repeat the colonoscopy.

Conversely, the United States Multi‐Society Task Force suggests that FIT‐positive patients who have recently undergone a colonoscopy should generally be offered a repeat colonoscopy.^8^ Kim *et al*. recently found a high risk of CRC in FIT‐positive patients who underwent colonoscopy in Korea.^13^ Furthermore, Chiu *et al*. reported that a higher fecal hemoglobin concentration was associated with the risk of postcolonoscopy CRC in a Taiwanese population‐based screening program.^14^

In this study, we investigated the correlation between the prevalence of advanced neoplasia or invasive cancer and fecal hemoglobin concentration in examinees who previously underwent a colonoscopy. Because we reported that FIT might be ineffective in patients with a history of colonoscopy within 5 years,^11^ we aimed to evaluate whether raising the cut‐off value of FIT would be useful to detect postcolonoscopy CRC in patients.

## Methods

### *Study design*

In this observational study, we retrospectively enrolled FIT‐positive patients who underwent colonoscopy through our opportunistic annual screening program from April 2010 to March 2017 at the Kyoto Second Red Cross Hospital, Japan. We examined the association between fecal hemoglobin concentration and prevalence of lesions. The primary end‐point was advanced neoplasia (defined as any tubular adenoma measuring ≥10 mm or with features of villous histology, high‐grade dysplasia, or invasive cancer). The secondary end‐point was invasive cancer. Advanced adenoma was defined as advanced neoplasia without invasive cancer. We recommended a colonoscopy if FIT results were positive in at least one of the two samples, regardless of whether the patients had undergone a colonoscopy. We excluded participants if they had received a colonoscopy within 6 months, had an unknown history of colonoscopy, or were aged <40 years. We divided patients into three groups based on whether they underwent colonoscopy previously (“no colonoscopy group” [those who never underwent colonoscopy previously], “past colonoscopy (≤5 years) group” [those who had undergone colonoscopy within 5 years], and “past colonoscopy (>5 years) group” [those who have undergone colonoscopy over 5 years ago]) and analyzed the relationship between fecal hemoglobin concentration and the prevalence of advanced neoplasia or invasive cancer in each group. Informed consent was obtained from all participants before colonoscopy, and the study protocol was approved by the Institutional Review Board of Kyoto Second Red Cross Hospital (Sp2018‐19).

### *FIT* *and cut‐off value*

The fecal samples were analyzed using the FOBIT Wako FIT kit (Fujifilm Wako Pure Chemical Industries, Osaka, Japan) with an annual 2‐day FIT method. We provided instructions to examinees on how to use the kit. In our opportunistic screening program, the cut‐off value applied was 30 ng/mL, which was lower than the usual cut‐off value (100 ng/mL) because our endoscopic center was able to support a sufficient number of colonoscopies.

### *Colonoscopy*

We used a modified split‐dose bowel preparation (magnesium citrate the day before and polyethylene glycol the morning of the colonoscopy). An enema was administered just before the procedure if the preparation was inadequate. All colonoscopies were performed or supervised by experienced endoscopists who were certified by the Japan Gastroenterological Endoscopy Society or its equivalent.

### *Data collection*

The sample size was determined by the number of cases enrolled in our hospital during the study period. Data were obtained from an endoscopy database, Solemio ENDO (Olympus Co., Tokyo, Japan). Indications for the colonoscopy, family history of CRC, history of colonoscopy, and colorectal polyps or cancers were recorded in the database shortly after the colonoscopy by an endoscopist by referring to the electric medical charts and questionnaires. For previously performed colonoscopies, we considered only those that were carried out in our hospital, whereas those performed at other hospitals were excluded as “unknown colonoscopy history” to avoid ambiguous interview recordings from the examinees. Both the fecal hemoglobin concentration data generated during the screening program and the endoscopy data extracted from the Solomio ENDO were linked using the hospital identification number in Excel (Microsoft Corporation, Washington, USA). The data of fecal hemoglobin concentration used were within 1 year from the date of colonoscopy. The higher fecal hemoglobin concentration of the 2 days was considered.

### *Statistical analysis*

The fecal hemoglobin concentration in the FIT was classified arbitrarily in four groups (30–100, 101–300, 301–500, and ≥501 μg/mL) and was compared to the detection rates of the lesions. The Cochran–Armitage test was used to examine the trends in the detection rate of advanced neoplasia in each group. We determined that the differences were significant if the calculated *P*‐value was less than 0.05. Furthermore, receiver operating characteristic (ROC) curves of FIT were plotted, and the area under the curve (AUC) value was calculated for each group. The Cochran–Armitage test was conducted using SAS statistical software version 9.4 (SAS Institute, Cary, NC, USA), and other statistical analyses were performed using IBM SPSS statistics version 22.0 (IBM Corp., Armonk, NY, USA).

## Results

**Figure 1:**
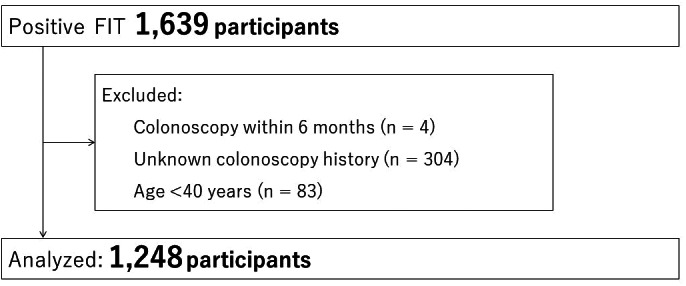
Study flow.

We assessed 1639 patients who underwent colonoscopy due to positive FIT results. Of these, 391 were excluded based on our exclusion criteria (colonoscopy within 6 months, *n* = 4; unknown colonoscopy history, *n* = 304; age <40 years, *n* = 83), and 1248 were analyzed (Fig. 1). The gender of the patients was 56.7% men (707/1248) and 43.3% women (541/1248). The mean age was 60.0 years old (range, 40–90). A first‐degree family history of CRC was identified in 9.1% (114/1248). There were 748 (59.9%), 198 (15.9%), and 302 (24.2%) patients in the no colonoscopy group, past colonoscopy (>5 years) group, and past colonoscopy (≤5 years) group, respectively (Table 1). In the past colonoscopy (>5 years) group, six (3.0%) and three (1.5%) patients had a history of advanced adenoma and invasive cancer, respectively. In the past colonoscopy (≤5 years) group, 13 (4.3%) and 5 (1.7%) patients had a history of advanced adenoma and invasive cancer, respectively.

**Table 1:** Patient characteristics

	No colonoscopy group (*n* = 748)	Past colonoscopy (>5 years) group (*n* = 198)	Past colonoscopy (≤5 years) group (*n* = 302)
Gender, *n* (%)			
Female	355 (47.5)	82 (41.4)	104 (34.4)
Male	393 (52.5)	116 (58.6)	198 (65.6)
Age (range), *n* (%)			
40–49	183 (24.5)	13 (6.6)	32 (10.6)
50–59	233 (31.1)	48 (24.2)	76 (25.2)
60–69	238 (31.8)	82 (41.4)	109 (36.1)
70–79	79 (10.6)	51 (25.8)	74 (24.5)
80–	15 (2.0)	4 (2.0)	11 (3.6)
History of colorectal lesions, *n* (%)			
Absent	NA	130 (65.7)	145 (48.0)
Adenoma	NA	45 (22.7)	105 (34.8)
Advanced adenoma	NA	6 (3.0)	13 (4.3)
Invasive cancer	NA	3 (1.5)	5 (1.7)
Unknown	NA	14 (7.1)	34 (11.3)
Family history of CRC, *n* (%)	68 (9.1)	24 (12.1)	22 (7.3)

CRC, colorectal cancer; NA, not applicable; *n*, number.

**Figure 2:**
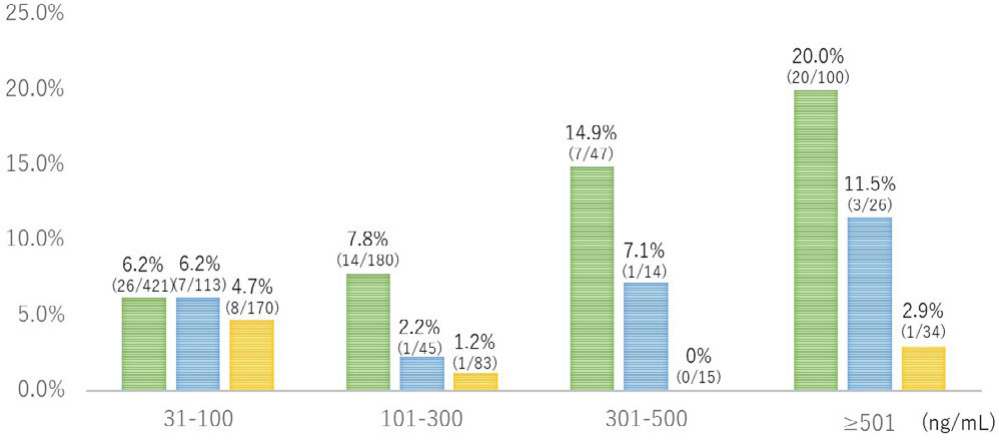
Relationship between fecal hemoglobin concentration and advanced neoplasia detection rates. The advanced neoplasia detection rate significantly increased with the fecal hemoglobin concentration in the no colonoscopy group, whereas no significant trend was observed in the past colonoscopy groups. Particularly in the past colonoscopy (≤5 years) group, the group with a lowest hemoglobin concentration had higher detection rate of lesions. 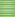, No colonoscopy group; 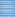, past colonoscopy (>5 years) group; 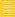, past colonoscopy (≤5 years) group.

The advanced neoplasia detection rates in the no colonoscopy group were 6.2% (26/421) at the fecal hemoglobin concentration of 30–100 ng/mL, 7.8% (14/180) at 101–300 ng/mL, 14.9% (7/47) at 301–500 ng/mL, and 20% (20/100) at >500 ng/mL (Fig. 2). The Cochran–Armitage test revealed that the advanced neoplasia detection rate significantly increased with the fecal hemoglobin concentration in the no colonoscopy group (*P* < 0.001). However, no significant trend was observed in the past colonoscopy (both >5 years and ≤5 years) group (*P* = 0.982). In the past colonoscopy (≤5 years) group in particular, the group with the lowest hemoglobin concentration had a higher detection rate of lesions (Fig. 2).

**Figure 3:**
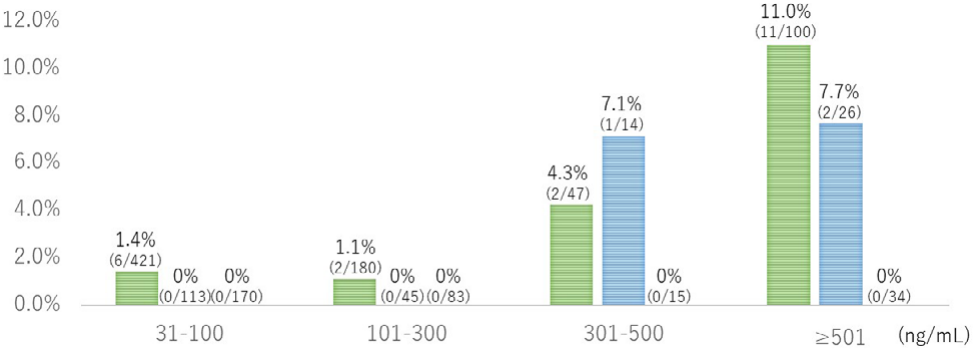
Relationship between fecal hemoglobin concentration and invasive cancer detection rates. Invasive cancer detection rate significantly increased with the fecal hemoglobin concentration in the no colonoscopy group, whereas no invasive cancer was detected in the past colonoscopy (≤5 years) groups. In the past colonoscopy (>5 years) group, several invasive cancers were detected in the groups with hemoglobin concentration over 300 ng/mL. 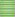, No colonoscopy group; 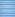, past colonoscopy (>5 years) group; 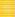, past colonoscopy (≤5 years) group.

Similarly, the invasive cancer detection rates in the no colonoscopy group significantly increased with the fecal hemoglobin concentration (*P* < 0.001; Fig. 3); however, no invasive cancer was detected in the past colonoscopy (≤5 years) group. In the past colonoscopy (>5 years) group, several invasive cancers were detected in the groups with hemoglobin concentration over 300 ng/mL.

**Figure 4:**
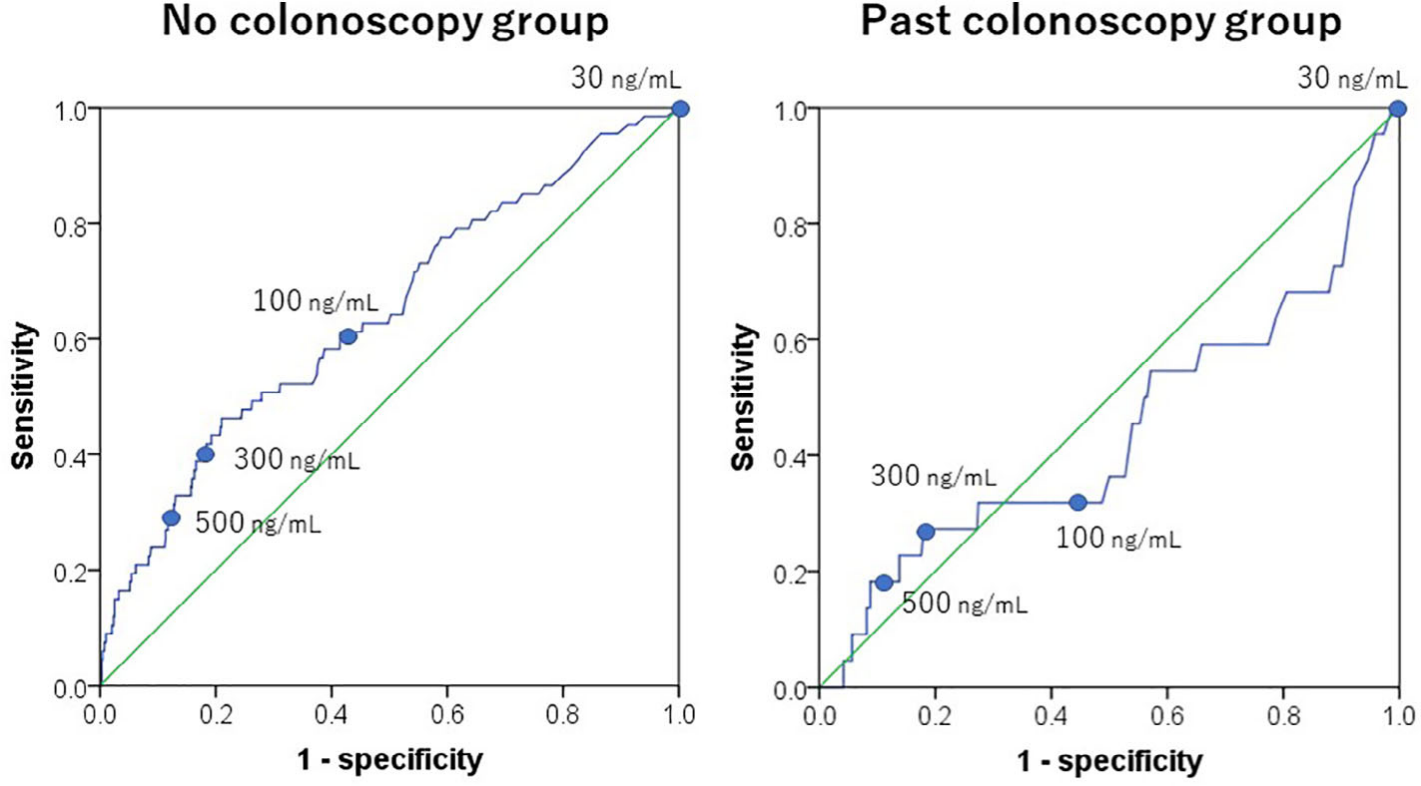
Receiver operating characteristic (ROC) curve of fecal immunochemical test. The area under the curve (AUC) values for advanced neoplasia were 0.641 (95% confidence interval [CI], 0.569–0.714) and 0.443 (95% CI, 0.302–0.584) for the no colonoscopy and past colonoscopy (>5 years and ≤5 years) groups, respectively.

Due to the small number of outcomes, we combined two past colonoscopy groups (≤5 years and >5 years) when drawing the AUC curve. The AUC values for advanced neoplasia were 0.641 (95% confidence interval [CI], 0.569–0.714) and 0.443 (95% CI, 0.302–0.584; Fig. 4) for the no colonoscopy and past colonoscopy groups, respectively.

## Discussion

In this study, we sought to evaluate whether postcolonoscopy colorectal neoplasms could be efficiently detected by raising the cut‐off value of FIT. However, the advanced neoplasia detection rate failed to show an association with increased fecal hemoglobin concentration in the past colonoscopy group. Furthermore, the AUC value of FIT was extremely low (<0.5) in subjects with a history of colonoscopy. Therefore, the detection of advanced neoplasia would be ineffective even if a more stringent cut‐off value was used.

Our results are not in line with the study by Chiu *et al*., who reported a significant association of fecal hemoglobin concentration with the risk prediction of postcolonoscopy CRCs.^14^ This difference could be attributed to the study design as the study by Chiu *et al*. was a population‐based study, in contrast to our single‐hospital, opportunistic‐screening program. Moreover, we used an annual 2‐day FIT method and a lower cut‐off value (30 ng/mL; 7.5 μg/g feces). Furthermore, split‐dose bowel preparation was used for all colonoscopies performed or supervised by experienced endoscopists. Therefore, we believe the quality level of colonoscopy in our hospital would be highly controlled. Our results indicated that repeated FIT might be ineffective if a high‐quality colonoscopy was ensured.

The results of our study also contradict those of the previous study by Kim *et al*., which found repeated FIT to be effective postcolonoscopy.^13^ We believe that their study might include many high‐risk patients, including those after polypectomy or surgery.^15^ In the present study, small samples of high‐risk patients were included as many of the examinees had no history of colorectal neoplasms or family history of CRC. Although repeated FIT postcolonoscopy could be effective in high‐risk patients, our results indicated that it might be ineffective in average‐risk patients following a colonoscopy, especially within 5 years.

However, our study indicates that advanced neoplasms were still detected in 10 of 302 FIT‐positive patients (3.3%) who underwent colonoscopy within 5 years. In this study, comparison with the FIT‐negative group could not be performed; thus, further studies are needed to determine whether or not FIT after endoscopy is effective. Consequently, it would be difficult to apply our results immediately to a population‐based CRC screening program. In Japan, an annual FIT is recommended for people aged 40 years or over even after a negative colonoscopy. This policy deviates from the European guidelines recommendation to repeat colonoscopy 10 years after a negative colonoscopy.^12^ Therefore, it would be necessary to conduct a multicenter study verifying the period in which repeat CRC screening is not necessary following a negative colonoscopy.

This study had several limitations. First, this included a single‐center retrospective observational study design. Because of the retrospective design, we could not assess the exact quality of past colonoscopies. Furthermore, exact risk stratification of the examinees could not be performed, although many of the patients were considered to be at average risk because many of the examinees had no history of colorectal neoplasms or family history of CRC. Second, we used advanced neoplasia as a surrogate end‐point instead of invasive cancer due to the small sample size of invasive cancers in our cohort. Finally, examinees with hemoglobin concentration <30 ng/mL were not included in this study as no colonoscopy was performed in these patients. Besides these limitations, our study showed that repeated annual FIT might be ineffective following a colonoscopy, especially within 5 years. However, even raising the stringency in cut‐off value of FIT could not detect advanced neoplasia after colonoscopy.

## Declaration of conflict of interest

The authors declare no conflicts of interest.

## Author Contributions

Takuji Kawamura contributed to study concept and design, acquisition of data, analysis and interpretation of data, and drafting of the manuscript. Takato Inoue, Ryo Shinomiya, Hiroaki Sakai, Kana Amamiya, Naokuni Sakiyama, Atsushi Shirakawa, Yusuke Okada, Kasumi Sanada, Kojiro Nakase, Koichiro Mandai, Azumi Suzuki, Mai Kamaguchi, Atsushi Morita, Kenichi Nishioji, and Koji Uno contributed to the acquisition of data. Kiyohito Tanaka contributed to the acquisition of data and technical support. Isao Yokota contributed to statistical review. Koji Uno, Masao Kobayashi, and Kenjiro Yasuda contributed to study supervision. All authors approved the final version of this manuscript.
